# A Synthesis of Algorithms for Multi-Jurisdiction Research in Canada.

**DOI:** 10.23889/ijpds.v7i3.1911

**Published:** 2022-08-25

**Authors:** Lisa Lix, Viktoriya Vasylkiv, Olawale Ayilara, Lindsey Dahl, Allison Poppel, Saeed Al-Azazi

**Affiliations:** 1 University of Manitoba

## Objectives

Validation of algorithms to identify health conditions (e.g., diabetes) or service use (e.g., high-cost users) in administrative data is time-consuming and expensive. Many algorithms are only assessed in a single jurisdiction, which may limit generalizability. Our study described the characteristics of multi-jurisdiction algorithms from a Canadian algorithm repository.

## Approach

We summarized algorithms captured in the open-access Algorithms Inventory developed by Health Data Research Network (HDRN) Canada. This inventory contains published algorithms identified through a series of systematic reviews of peer-reviewed research. Algorithms included in the inventory were validated or assessed for feasibility of implementation in two or more provinces/territories; they encompass measures of population health, health service use, and determinants of health. Descriptive statistics were used to characterize the study data on such features as year and discipline of the study journal, algorithm topic area, jurisdictions included in the study, validation source data, and algorithm elements (i.e., diagnosis codes).

## Results

The HDRN Canada Algorithms Inventory currently contains 166 algorithms from 63 published articles. The majority of articles were published in 2010 or later (89%) and more than half (56%) of the articles were found in journals with a clinical focus. Feasibility studies (79%) were conducted more often than validation studies (21%). Most algorithms used data from the provinces of British Columbia, Manitoba, Ontario, and Nova Scotia. The majority of algorithms (72%) measured population health concepts, such as chronic physical health conditions (63%; e.g., hypertension) and mental health conditions (14%; e.g., depression). Algorithms about the determinants of health (17%) mostly focused on measures of socioeconomic status (37%) derived from census data. Multi-jurisdiction algorithms about health service use were least common (11%).

## Conclusion

This synthesis revealed few Canadian multi-jurisdiction validation studies have been conducted and not all provinces/territories are equally represented. New validation studies, particularly about health service use and determinants of health, will increase the consistency and accuracy of Canadian research. Reusing published algorithms from this inventory will facilitate research reproducibility.

